# Refining Pathways: A Model Comparison Approach

**DOI:** 10.1371/journal.pone.0155999

**Published:** 2016-06-01

**Authors:** Giusi Moffa, Gerrit Erdmann, Oksana Voloshanenko, Christian Hundsrucker, Mohammad J. Sadeh, Michael Boutros, Rainer Spang

**Affiliations:** 1 Department of Statistical Bioinformatics, Institute of Functional Genomics, University of Regensburg, Regensburg, Germany; 2 Division of Signaling and Functional Genomics, German Cancer Research Center (DKFZ) and Department of Cell and Molecular Biology, Faculty of Medicine Mannheim, Heidelberg University, Heidelberg, Germany; Rutgers, the State Univesity of New Jersey, UNITED STATES

## Abstract

Cellular signalling pathways consolidate multiple molecular interactions into working models of signal propagation, amplification, and modulation. They are described and visualized as networks. Adjusting network topologies to experimental data is a key goal of systems biology. While network reconstruction algorithms like nested effects models are well established tools of computational biology, their data requirements can be prohibitive for their practical use. In this paper we suggest focussing on well defined aspects of a pathway and develop the computational tools to do so. We adapt the framework of nested effect models to focus on a specific aspect of activated Wnt signalling in HCT116 colon cancer cells: Does the activation of Wnt target genes depend on the secretion of Wnt ligands or do mutations in the signalling molecule *β*-catenin make this activation independent from them? We framed this question into two competing classes of models: Models that depend on Wnt ligands secretion versus those that do not. The model classes translate into restrictions of the pathways in the network topology. Wnt dependent models are more flexible than Wnt independent models. Bayes factors are the standard Bayesian tool to compare different models fairly on the data evidence. In our analysis, the Bayes factors depend on the number of potential Wnt signalling target genes included in the models. Stability analysis with respect to this number showed that the data strongly favours Wnt ligands dependent models for all realistic numbers of target genes.

## Introduction

Cellular signalling pathways like the Wnt pathway can be represented as networks modelling signal propagation, amplification and modulation of multiple molecular interactions. An important objective of systems biology is learning and adjusting the network topologies of pathways from experimental data.

During signal propagation, upstream components of the pathway control the activation of downstream components and, indirectly, the expression of target genes. Perturbations of signalling components affect the regulation of target genes and these gene expression changes reflect the topology of the pathway: Blocking an upstream component like a molecule of a receptor complex automatically blocks the activation of downstream components like signalling mediators or transcription factors. In fact, we use this concept as a definition of upstream/downstream relationships in pathways. With this definition, upstream components change the expression of more target genes then downstream components. Nested effects models (NEMs) implement this concept into an algorithm for inferring pathway topologies [1–3]. They have been successfully applied to analyse LPS-mediated signalling in Drosophila cells [1], B-cell receptor signalling in human BL2-cells [4], cellular decision making in early murin embryonic stem cells differentiation [3], the yeast mediator complex [5], rhinovirus infection mechanisms [6], or gene regulatory interaction networks in C. elegans [7].

A new perturbation data set can support the current working model of a pathway or it can falsify it. NEMs are statistical tools designed to analyze such data. They predict a topology that can be compared to the current model. To apply the basic NEM algorithm, we must specify all signalling components in a pathway, silence or inhibit all of them, monitor all target genes, and provide this data as input to the algorithm, which returns the best scoring pathway topology. There can be many obstacles to this enterprise: Lists of a pathway’s signalling components and target genes are incomplete, or they differ between references. It might be too expensive or even impossible to perform all perturbation experiments. Even if we accomplish all experiments, the pathway with all its fine details can be so large that computing its topology becomes prohibitive. We argue that in these cases resolving the full pathway is too ambitious.

A working model is falsified, if a specific aspect of the topology like an edge is in clear conflict to data. For instance, a pathway model that does not include cross-talk between two parallel branches of a pathway, while the data cannot be explained without such cross-talk. The question whether there is cross talk reduces to a single bit of information: a *yes* or a *no*. The basic NEM algorithm will provide a complex topology. A topology that with limited input data is very questionable. However, if we combine nested effects models with statistical principles like model comparison and stability analysis we can address the questions directly focussing on the single bit of information desired. In a statistical case study on Wnt signalling in HCT116 colon cancer cells, we show how properties of the pathway’s topology can be studied using only a few perturbation assays and easily feasible computations.

More than 85% [8] of sporadic colorectal cancers (CRC) harbour mutations in proteins which regulate the canonical Wnt signalling pathway, like the adenomatous polyposis coli gene (*APC*) or the *CTNNB1/β-catenin* gene. Upon activation of the pathway, the destruction complex dissociates, *β*-catenin accumulates and translocates to the nucleus. There *β*-catenin acts as a transcriptional coactivator for TCF transcription factors (including *TCF7L2*) leading to the regulation of target genes such as *AXIN2*. Many cells produce Wnt ligands that signal in an autocrine or paracrine manner. Wnt ligands secretion depends on the Wnt-specific secretion factor Evi/Wls. The cell line HCT116 has a mutated CTNNB1 gene, which encodes the signalling protein *β*-catenin. We focus on the following question: Does the activation of Wnt target genes still depend on the secretion of Wnt ligands (a) or do the CTNNB1 mutations make this activation independent from them (b)?

We framed the binary question into two competing classes of pathway models: Models that depend on Wnt ligand secretion versus those that do not. The model classes translate into restrictions of the pathway topology space. Since Wnt dependent models are more flexible than Wnt independent models, we need to account for the extra complexity in our comparison of the two models. Bayes factors do so and fairly compare the data evidence for competing models. In our analysis, the Bayes factors depended on the number of potential Wnt signalling target genes included in the models. Stability analysis showed that the data strongly favours Wnt ligand dependent models for all (realistic) numbers of Wnt target genes.

For this analysis, we performed RNA interference experiments in HCT116 cells where we silenced just four genes: *EVI/WLS*, *APC*, *CTNNB1/β-catenin*, and *TCF7L2*. We monitored changes in target gene expression by RNAseq. Details about the experimental protocols are given in the appendix. This previously unpublished dataset is available at the ArrayExpress database with accession number E-MTAB-651. Supplementary material for full reproducibility of the analysis is available on github at https://github.com/annlia/featureNEM.

## Methods

### Restrictions on the topology of a signalling network can be sustained or rejected using Bayes factors on topology classes

Let *f* be an arbitrary feature of a topology, we denote by $C_{f}$ the class of all topologies that exhibit feature *f* and by $C_{\bar{f}}$ the complimentary class of topologies that do not have it. Bayes factors [9] are the classical Bayesian tools for model comparison, where we wish to quantify the evidence which the data ***D*** provides in favour of a model $M_{1}$ relative to a different model $M_{0}$. The Bayes factor *B*_10_ of model $M_{1}$ versus $M_{0}$ is defined as the ratio between the posterior and the prior odds

$$B_{10}=\frac{P\left(M_{1}|D\right)/P\left(M_{0}|D\right)}{P\left(M_{1}\right)/P\left(M_{0}\right)}\equiv \frac{P(D|M_{1})}{P(D|M_{0})}$$

which is the ratio between the models’ marginal likelihoods. The Bayes factor provides more protection against over-fitting compared to log-likelihood ratios, since the parameters *θ*_*m*_ are integrated out rather than maximised

$$P\left(D|M_{m}\right)=\int_{\Theta_{m}}P\left(D|\theta_{m},M_{m}\right)P\left(\theta_{m}|M_{m}\right)d\theta_{m},\;m=0,1$$

If the models consist of topology classes: a class $C_{f}$ of topologies with the feature *f* and its complement class $C_{\bar{f}}$, the Bayes factor is

$$B_{f\bar{f}}=\frac{P(D|C_{f})}{P(D|C_{\bar{f}})}$$

The models in each class will be sets of NEMs. To evaluate the Bayes factors we therefore need the mathematical treatment of their marginal likelihoods *P*(***D***|*C*_*f*_), which we turn to next.

### Quantifying the evidence for NEM structural features via Bayes factors

**Fig 1:**
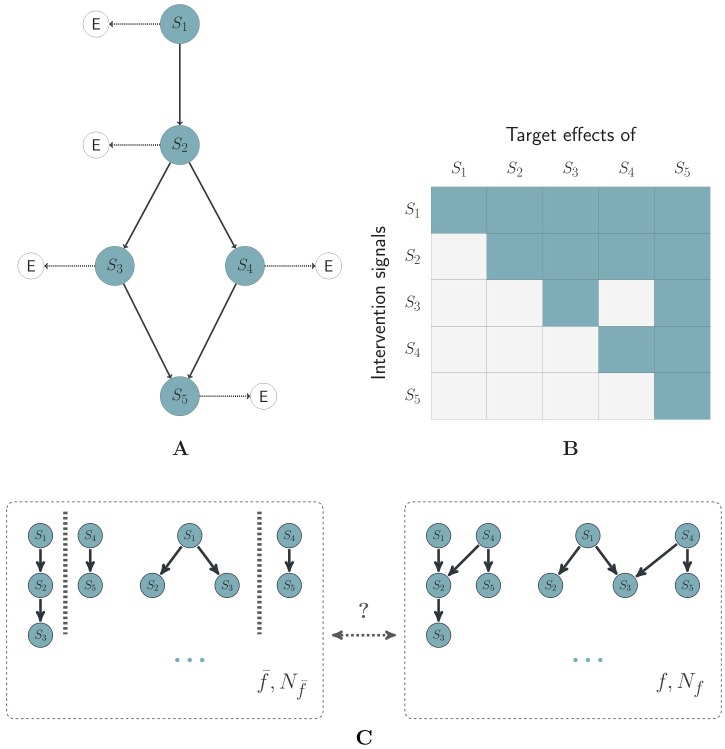
Nested effect models and structural features. An example of a NEM network, its expected observation pattern and structural features. **A** shows a signalling network including S- and E-genes. **B** shows the corresponding expected downstream effects of E-genes that are attached to S-genes (columns), if the S-genes in the rows are blocked. **C** shows classes of topologies that share a specific feature. In this case the distinguishing feature is the existence of an edge that connects the sub-networks formed by (*S*_1_, *S*_2_, *S*_3_) and (*S*_4_, *S*_5_). The topologies on the right have this feature while those on the left do not.

NEMs are probabilistic models for reverse engineering signalling pathways [1]. They represent pathways by directed graphs with distinct nodes for the signalling proteins (S-genes) and for the effected target genes (E-genes) that change expression in response to perturbations of S-genes. A NEM predicts that an E-gene changes expression when blocking the S-gene *S*, if and only if it is connected to *S* or to a descendent of *S*. Fig 1 gives examples for a nested effect model (A) and the expected data pattern it encodes (B).

In the Bayesian framework of [1] full network topologies are scored by their marginal posterior probability given the data ***D***, while integrating out the parameters which describe how E-genes attach to S-genes. Topologies with the same transitive closure cannot be distinguished on the basis of data because they are score equivalent [1]. Let Φ = (*ϕ*_*ij*_) be the adjacency matrix of a transitively closed network, *θ* = (*θ*_*l*_) be the attachment parameters, such that *θ*_*l*_ = *k* if *E*_*l*_ is attached to *S*_*k*_, and ***D*** = (*d*_*lk*_) a data matrix whose elements quantify the effect observed for *E*_*l*_ when ‘blocking’ *S*_*k*_. The network posterior probability is

$$P\left(\Phi |D\right)=\frac{P(D|\Phi )P(\Phi )}{P(D)}$$

The marginal likelihood *P*(***D***|Φ) is obtained by summing over *θ*

$$P\left(D|\Phi \right)=\sum_{\theta }P\left(D|\theta ,\Phi \right)P\left(\theta |\Phi \right)$$ (1)

where *P*(*θ*|Φ) is a prior distribution for *θ* and is assumed to be independent of the network structure Φ, such that it can be written as *P*(*θ*).

Defining ***D***_*l*_ as the *l*-th row of the data matrix, or in other words the vector of observations corresponding to E-gene *E*_*l*_, the likelihood decomposes as

$$P\left(D|\Phi ,\Theta \right)=\prod_{l=1}^{L}P\left(D_{l}|\Phi ,\theta_{l}\right),\;P\left(D_{l}|\Phi ,\theta_{l}\right)=\prod_{k=1}^{K}P\left(d_{lk}|\Phi ,\theta_{l}\right)$$ (2)

Different likelihood models *P*(*d*_*lk*_|Φ, *θ*_*l*_) exist for discrete [1] and continuous data [10]. Expression profiling data is better described as continuous, so we chose a Bayesian version of the continous data likelihood. Expression changes of E-genes were provided as posterior probabilities *p*_*lk*_, where *l* refers to an E-gene and *k* to a perturbation. We used the Bayesian linear modelling implemented in the limma package [11–13] to calculate these posterior probabilities. Finally, network topologies are scored by the marginal likelihood in Eq (1) where the terms in the likelihood Eq (2) are defined as

$$P\left(d_{lk}\right)=\left\{\begin{array}{cc} p_{lk} & \;\text{if}\;\Phi \;\text{predicts}\;\text{an}\;\text{effect}\\ 1-p_{lk} & \text{otherwise} \end{array}\right.$$

Fig 1 shows an example of two classes of topologies $C_{f}$ and $C_{\bar{f}}$ that are distinguished by a feature *f*. Let N be the total number of admitted topologies on the set of S-genes and *N*_*f*_ and $N_{\bar{f}}$ the numbers of topologies in $C_{f}$ and $C_{\bar{f}}$ respectively. The Bayes factor for class $C_{f}$ versus $C_{\bar{f}}$ can be written as

$$\frac{P(D|C_{f})}{P(D|C_{\bar{f}})}=\frac{\sum_{n=1}^{N}P\left(D,\Phi_{n}|C_{f}\right)}{\sum_{n=1}^{N}P\left(D,\Phi_{n}|C_{\bar{f}}\right)}=\frac{\sum_{n=1}^{N}P\left(D|\Phi_{n},C_{f}\right)P\left(\Phi_{n}|C_{f}\right)}{\sum_{n=1}^{N}P\left(D|\Phi_{n},C_{\bar{f}}\right)P\left(\Phi_{n}|C_{\bar{f}}\right)}$$ (3)

Given the topology Φ_*n*_ the marginal likelihood of the data ***D*** is independent of the class *C*_*m*_ to which the topology belongs

$$P\left(D|\Phi_{n},C_{m}\right)\equiv P\left(D|\Phi_{n}\right)$$

for all *n* ∈ {1, …, *N*} and for $m=f,\bar{f}$. The prior distribution $P(\Phi_{n}|C_{m})$ of the network topologies is assumed to be uniform within each class

$$P\left(\Phi_{n}|C_{m}\right)=\frac{1}{N_{m}}I_{\left\{\Phi_{n}\in C_{m}\right\}}$$

for $m=f,\bar{f}$, with *I* the indicator function. The Bayes Factor then reduces to

$$\frac{P(D|C_{f})}{P(D|C_{\bar{f}})}=\frac{N_{\bar{f}}}{N_{f}}\frac{\sum_{\Phi_{n}\in C_{f}}P\left(D|\Phi_{n}\right)}{\sum_{\Phi_{n}\in C_{\bar{f}}}P\left(D|\Phi_{n}\right)}$$ (4)

where we can see that the class $C_{f}$ is penalised by the ratio $N_{\bar{f}}/N_{f}$ if it allows for more topologies than $C_{\bar{f}}$.

Which of the two classes $C_{f}$ and $C_{\bar{f}}$ describes the data better? If $C_{f}$ is larger then $C_{\bar{f}}$ does the data support this extra complexity of the class? The Bayes factor helps to answer exactly these questions. If the Bayes factor exceeds 1, the evidence favours including feature *f* in the working model of a pathway, otherwise *f* should not be part the model. More refined inference comes from including prior beliefs on the two model classes and considering the ratio of posterior probabilities.

## Results

### Perturbations of Wnt signalling in colon cancer HCT116 cells

We here describe a case study of a focused pathway analysis based on NEMs. To this end, we investigate canonical Wnt signalling in colon cancer HCT116 cells. These cells carry a heterozygous one amino acid (*δ*SERer45 [14, 15]) deletion in the *β*-catenin protein [16] which has been associated with constitutively active canonical Wnt signalling [15]. Recently, it was proposed that independent of constantly active mutation of *β*-catenin upstream components (like Evi/Wls), Lrp5/6 and Dvls regulate the canonical Wnt pathway [17].

**Fig 2:**
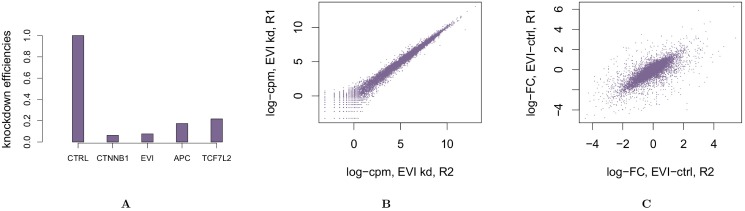
Experiments’ efficiency and reproducibility. Panel **A** reports the relative expression of the direct siRNA targets with respect to the control in the corresponding experiment. log-cpm measured in the two replicates of the EVI silencing experiment are shown in panel **B**. Panel **C** shows the log fold changes between the cpm measured in two pairs of experiment, namely EVI silencing and CTRL.

We depleted signalling molecules at different levels of the Wnt pathway using RNA interference (RNAi). Specifically, we silenced EVI/WLS, APC, CTNNB1/*β*-catenin, and TCF7L2 by RNAi. Changes in the expression of Wnt target genes were monitored using RNAseq. In addition HCT116 cells were treated with a non-targeting siRNA as a control. All experiments were replicated twice, with the exception of TCF7L2, for which only one biological replicate was available. In addition, two samples of HCT116 cells were treated with a nonsense siRNA and profiled as control. Silencing efficiency of the genes was confirmed by qPCR (data not shown) and showed efficiencies above 80% (Fig 2A). On average 37 million sequence reads were generated per knock-down. Reproducibility of the log-cpm value between the two replicates of the same experiment is shown in Fig 2B for the EVI/WLS knock-down. Reproducibility of log-cpm values translates into reproducibility of log fold changes of cpm values, when comparing perturbations to controls (Fig 2C). Similar reproducibility could also be observed for the other experiments. The fold changes also matched the qPCR knock-down results. For example, RNAi depletion of CTNNB1/*β*-catenin led to a log fold change of the CTNNB1/*β*-catenin gene of -3.98, and a log fold change of -3.71 in the Wnt target gene AXIN2, whereas non-related genes such as ACTB were not affected (S1 Fig). The RNAseq data also confirmed the presence of the mutated CTNNB1/*β*-catenin allele, which expressed at higher levels than the wild-type allele (S1 Fig). Indeed, we could not detect any phosphorylation of S45 which is deleted in the mutated allele, suggesting that in HCT116 mutated CTNNB1/*β*-catenin is more abundant than in the wild-type.

**Fig 3:**
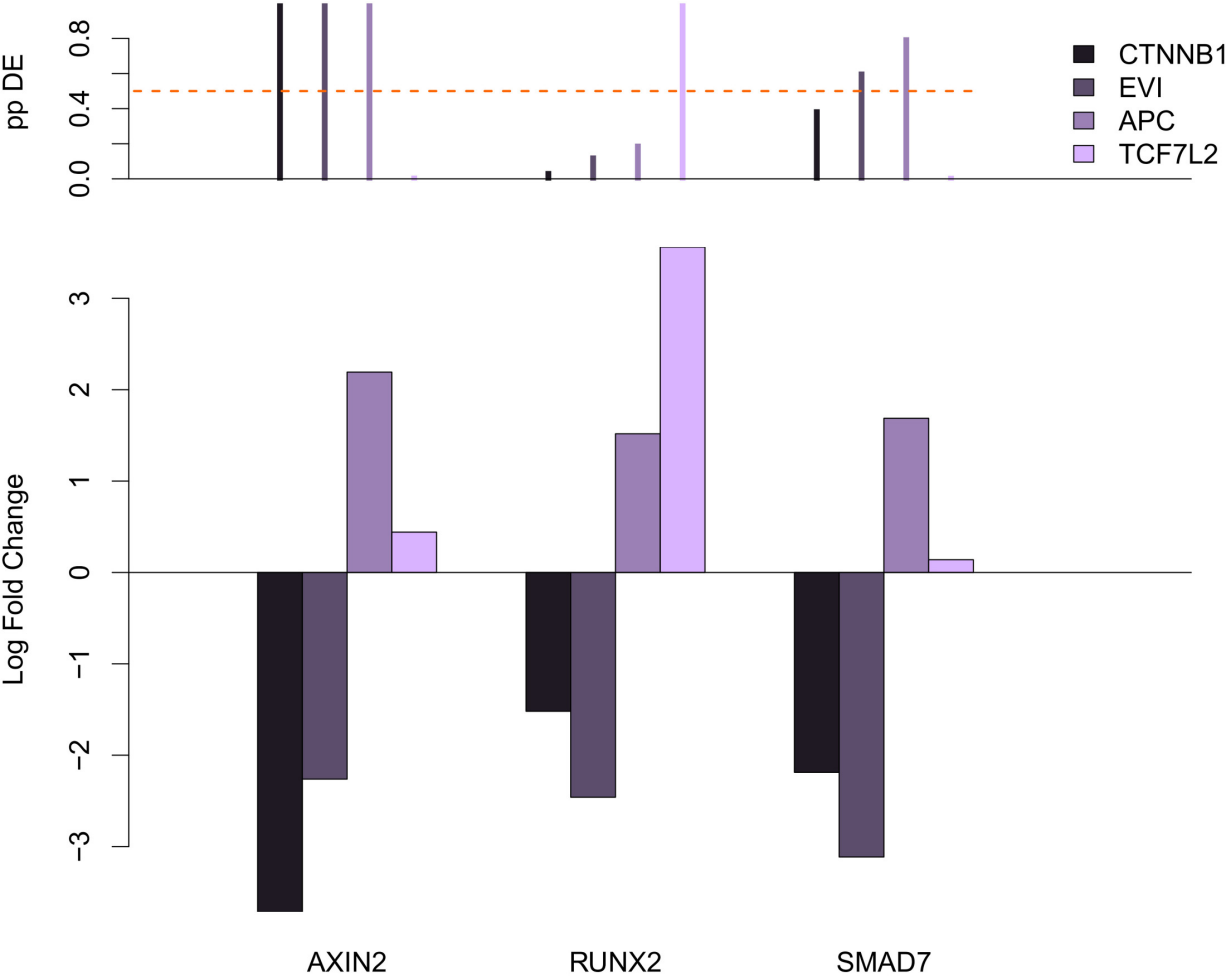
Expression of Wnt target genes. Comparison of gene expression profiles after siRNA mediated knock-down of Wnt pathway components show high similarity between *EVI/WLS* and downstream positive pathway regulators. The top panel shows the posterior probability of differential expression in the experiments, with the dashed line marking probability.5. There is strong evidence for differential expression of the canonical target gene AXIN2 when silencing *CTNNB1/β-catenin*, *EVI/WLS* and *APC*. RUNX2 and SMAD7 show a similar pattern of opposite regulation after knock-down of *APC* and *CTNNB1/β-catenin*, but while for SMAD7 the posterior probability of differential expression are still above a half for the *EVI/WLS* and *APC* knock-down, the probabilities for RUNX2 are below, which translates into reduced evidence for reproducibility of the fold changes.

We preprocessed the count data using the voom function of the limma package [13] and calculated fitted Bayesian linear models to the data using the limma package [11]. From these models we calculated the posterior probability that the expression of a gene is affected by the knock-down again using limma. For the direct targets of the siRNAs the posterior probabilities of change were all estimated as effectively 1. A probability of nearly 1 was also obtained for *AXIN2* under depletion of *CTNNB1/β-catenin*, while a probability well below.01 was estimated for the non Wnt target *ACTB* under the same intervention. Fold changes and corresponding posterior probabilities for a number of well known Wnt targets are shown in Fig 3.

**Fig 4:**
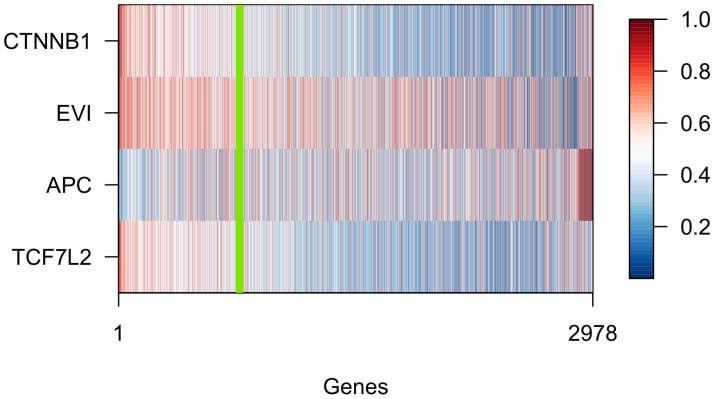
Posterior probabilities of differential expression. Heat-map of the posterior probabilities of differential expression in the silencing experiments. Only genes which have a posterior probability larger than .5 in at least one of the knock-down experiments are shown. The green line leaves about 750 genes on its left. The pattern there shows that the majority of those genes respond not only to intervention on *CTNNB1/β-catenin*, but also to *EVI/WLS*.

Transcriptome wide downstream effects are outlined in Fig 4 which shows a heat map of posterior probabilities for all four knock-downs and 2978 genes. Red corresponds to high probabilities of expression change and blue to virtually zero probability. All 2978 genes showed a high probability in at least one condition. The genes to the left of the green line reacted to both *CTNNB1/β-catenin* and *EVI/WLS* knock-downs. If we considered them all as bona fide Wnt target genes, the heat map would already give compelling evidence that the activation of Wnt target genes in fact depends on Evi/Wls and thus on Wnts secretion. Interestingly, most of these genes also responded to blocking TCF7L2 but only few of them to APC. To the right of the green line are some genes that are blue in the *EVI/WLS* row but red in the *CTNNB1/β-catenin* row. They respond to *CTNNB1/β-catenin* but not *EVI/WLS*. Nevertheless, they are a small minority.

### A focused analysis of Wnt signalling in HCT116 cells using Bayes factors

We now examine the descriptive arguments of the previous section with sound statistical inference. Our leading question is: Does the activation of Wnt target genes in HCT116 cells depend on the secretion of Wnt ligands (a) or do the CTNNB1 mutations make this activation independent from them (b)? And to answer this question we rely on perturbation data for four proteins *EVI/WLS*, *APC*, *CTNNB1/β-catenin*, and *TCF7L2*. The mutated gene CTNNB1 encodes for *CTNNB1/β-catenin*. Hence, the first two proteins (*EVI/WLS*, *APC*) operate upstream of the mutated protein, the third protein is mutated, and the last protein (*TCF7L2*) operates downstream of the mutated protein. All four proteins together constitute only a tiny fragment of the Wnt pathway.

We represented the two competing models (a) and (b) by classes of topologies. For the Wnt independent topologies (b) we requested that there are no edges connecting (*EVI/WLS* and *APC*) with (*CTNNB1/β-catenin* and *TCF7L2*). In contrast, for the Wnt dependent topologies (a) we requested that there must be at least one edge between the two groups of proteins. There were more topologies in the Wnt dependent class making the dependent model more complex and more flexible. A Wnt dependent topology fitted the data best. But was this simply because there are more of them, or did the data justify this additional model complexity? We had translated our initial biological question into a statistical model comparison problem. We chose Bayes factors to address it. The computational implications were already addressed in the method section.

A practical difficulty emerged when it came to naming all Wnt target genes. In our model they played the role of E-genes. Some canonical Wnt target genes, like AXIN2, SMAD7 and MYC, are well established; but there is no consensus on a complete list of Wnt targets. Most likely, the Wnt pathway regulates different target genes in different cellular systems. We decided to use our own RNAseq data to screen for potential Wnt targets in HCT116 cells. To this end, we ranked all genes by the maximum posterior probability across the four perturbations. For a cut-off *λ* we included all genes with a score above *λ*. This reduced the E-gene selection problem to a parameter calibration problem. Recalling Eq (2) we saw that determining the number of E-genes was crucial because this number drives the right hand product of conditional probabilities. So, how many Wnt target genes are there in HCT116 cells? The Nusse lab currently names 30 genes as Wnt targets in human colon cancer on their *The Wnt Homepage* resource http://web.stanford.edu/group/nusselab/cgi-bin/wnt/target_genes. All of them are backed up by publications. This list does not claim to be complete. In a more data driven approach we relied on our posterior probabilities and cut the list at a threshold of 95%. This gave us 1483 potential target genes with high support from data. In fact, determining the correct number of Wnt target genes in HCT116 cells might be a very hard problem, and not even well defined. Fortunately, stability analysis showed that we do not need an exact solution.

**Fig 5:**
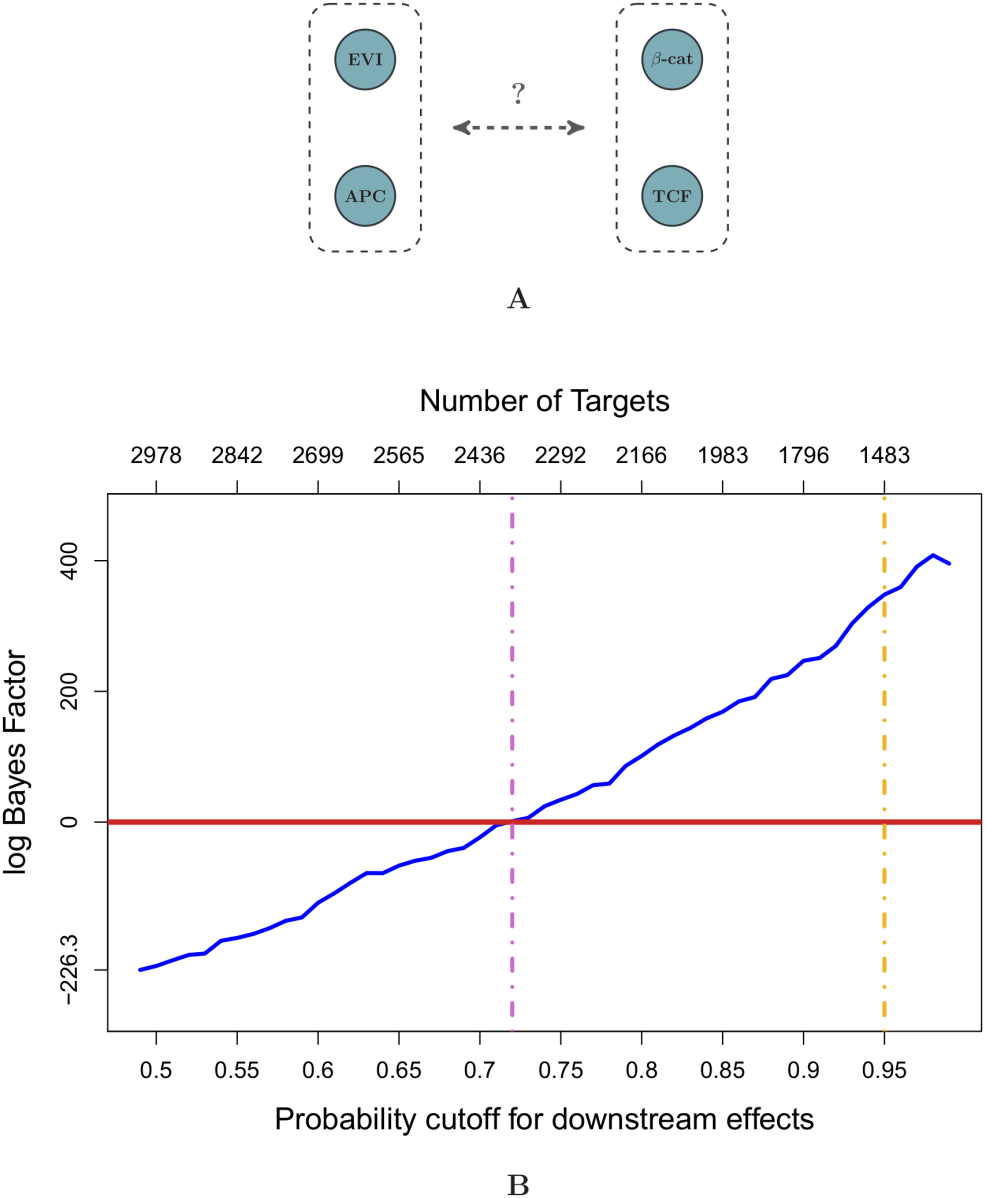
Model comparison for network structural features. Panel **A** is a schematic representation of the two topology classes compared. Panel **B** summarizes the main result of our statistical case study. Log Bayes Factors were obtained from NEMs including different numbers of potential target genes (x-axis, top), which are included according to a cut-off on the posterior probability that a gene is affected for at least one perturbation. Positive log Bayes factor reflect evidence in favour of a Wnt secretion dependent model. The dashed purple line indicates the smallest cut-off that still favours this topology class. A cut-off of 95% posterior probability is marked by the yellow dashed line.

We run the Bayes factor analysis for the full range of cut-offs between 0 and 1. Fig 5B plots the log Bayes factor against the number of E-genes used to calculate it. In the context of our initial question: Does the activation of Wnt target genes in HCT116 cells still depend on the secretion of Wnt ligands (a) or do the CTNNB1 mutations make this activation independent from them (b)? Positive log Bayes factors are evidence in favor of (a) while negative values support (b). We considered values above 10 or below -10 as strong evidence as they correspond to odds ratios exceeding 1:1000. If we used the 1483 E-genes corresponding to a 95% posterior probability cut-off we reached log Bayes factors much higher than 10 (dashed yellow line). If we used only the top 200 or the top 30 genes the evidence in favour of a Wnt secretion dependent model was even stronger. In fact, any model based on less then 2200 potential target genes endorsed Wnt secretion dependence (dashed purple line). This number greatly exceeds any number of direct Wnt target genes reported so far. With even lower cut-offs, we believe that noise dominated the models. In summary, although we do not know how many Wnt target genes there are in HCT116 cells, we could confidently answer our leading question. Our answer is (a): The activation of Wnt target genes in HCT116 cells depends on the secretion of Wnt ligands. All models with a realistic number of E-genes consistently answered (a).

Code and data for reproducing the plot of Fig 5B are provided as supplementary material.

## Discussion

In the context of a statistical case study we illustrated here how NEM modelling can be combined with model comparison and stability analysis to analyse complex pathways with limited data. We developed the computational implications of Bayes factor analysis in the context of NEM modelling with restricted topology classes. In the case study we were confronted with the statistical question on whether a perturbation data set justified the refinement of an existing simpler pathway model. By allowing for additional edges the pathway model became more complex, and naturally fitted data better. The Bayes factors penalized the additional model complexity but nevertheless strongly endorsed the more complex model. A complication emerged when we had to decide which and how many genes to include in the analysis. We overcame the problem by stability analysis. No matter how many genes we included, model comparison gave the same answer: The more complex model is appropriate.

We believe that strategies similar to that used in the case study can greatly improve the scope of problems in which NEM modelling can be applied. First, it is common that pathway features rather then complete pathways are under study. Second, it is also common that perturbation data is limited and does not cover all components of a pathway. Third, models of full pathways involve many parameters that need to be jointly estimated from data requiring very high numbers of repeated experiments to control the variance of the estimators. The variance of a single estimated binary parameter is easier to control. To do so, Bayesian model comparison is our method of choice. It can be used to focus analysis on a defined aspect of a pathway, it does not require data covering the full pathway, and, as shown in our case study, it provides strong collective evidence by joining individual evidence from many target genes even with small data sets.

To date, model comparison is not the standard approach to network analysis in biology. Most algorithms bank on a maximum likelihood approach, or on heuristic scoring systems. Maximum likelihood is prone to over-fitting. There is an eminent danger of deriving pathway models that are too complex. In the context of Gaussian graphical models this has been addressed for example by likelihood penalisation [18], or shrinkage [19]. For NEM no such approach existed to date. Bayes factors address the over-fitting problem. Eq (4) highlights the intrinsic penalty for larger topology classes.

In our understanding, all current working models of signalling pathways are abstractions that cannot live up to the true complexity of cellular processes. There is always data that cannot be explained by them. Pathway models focus on the dominant mechanisms of a pathway, those that are indispensable to understand it. If new data emerges that is in contradiction to an established pathway model, there is the inevitable question whether the new data provides sufficient evidence justifying a refinement of the pathway model. Especially, because every refinement comes with increasing complexity, that makes a pathway more difficult to understand. Model comparison addresses exactly this problem.

In our case study on the Wnt pathway we could sustain the proposition that the activation of Wnt target genes in colon cancer cells depends on Wnts secretion, at least for HCT116 cells. This result has been previously reported and endorsed by a series of biochemical experiments [17]. Our modelling adds a new aspect to the previous work. We analyse Wnt signalling controlled gene regulation on a transcriptome wide level. Surprisingly, we observed more than a thousand genes that changed expression to knock-downs of Wnt signalling components at a 95% posterior probability level. This included both activated and repressed genes suggesting that the Wnt pathway directly or indirectly affects many cellular processes, maybe even more than known today.

A limitation of the analysis in our study is that conclusions can only be drawn for the investigated HCT116 cells lines and not for colon cancer in general. We cannot exclude effects from the culturing or that there are colon cancers where the Wnt pathway operate in a Wnt secretion independent mode. To further uncover the important mechanisms in the Wnt pathway and colon tumour development, a comparative study of a diverse set of tumorous, as well as non-tumorous, colon primary cells would therefore be highly valuable. From such data, one could then refine the pathway inference and draw more generalised conclusions again utilising the general framework of network model comparison developed in this manuscript.

## Supporting Information

S1 Code Reproducible analysis. For the sake of allowing full reproducibility the complete code for the analysis presented in the manuscript is available on github at https://github.com/annlia/featureNEM.
(PDF)

S1 Protocols Protocols. Details about the experimental protocols are given in the supplementary material.
(PDF)

S1 Fig Sequence reads. Examples about the distribution of the sequence reads, with a quantification of the expression of both *β*-catenin alleles (mutated and wild-type) in HCT116 cells are reported in the supplementary material.
(PDF)

S2 Fig Phosphorylation. Results from Western blot analysis are reported in the supplementary material.
(PDF)

S1 Alternative Analysis No-CONAN analysis. Details about the No-CONAN approach.
(PDF)
