# Supplementary material for: Refining Pathways: A Model Comparison Approach
Source: PLoS One. 2016 Jun 1;11(6):e0155999. doi: 10.1371/journal.pone.0155999 (PMC4889067; doi:10.1371/journal.pone.0155999)
Supplement: S1 Code — For the sake of allowing full reproducibility the complete code for the analysis presented in the manuscript is available on github at https://github.com/annlia/featureNEM. (PDF) [file pone.0155999.s001.pdf]

Supplement - Refining pathways: a model comparison approach

Giusi Moffa[1, *], Gerrit Erdmann[2], Oksana Voloshanenko[2], Christian Hundsrucker[1], Mohammad J. Sadeh[1], Michael Boutros[2], Rainer Spang[1]

1 Department of Statistical Bioinformatics, Institute of Functional Genomics, University of Regensburg, Germany
2 Division of Signaling and Functional Genomics, German Cancer Research Center (DKFZ) and Department of Cell and Molecular Biology, Faculty of Medicine Mannheim, Heidelberg University, Germany

*** giusi.moffa@gmail.com**

S1 Code

The complete code for the analysis presented in the manuscript is available on github at `https://github.com/annlia/featureNEM`
