# Supplementary material for: Refining Pathways: A Model Comparison Approach
Source: PLoS One. 2016 Jun 1;11(6):e0155999. doi: 10.1371/journal.pone.0155999 (PMC4889067; doi:10.1371/journal.pone.0155999)
Supplement: S1 Protocols — Details about the experimental protocols are given in the supplementary material. (PDF) [file pone.0155999.s002.pdf]

Supplement - Refining pathways: a model comparison approach

Giusi Moffa[1, *], Gerrit Erdmann[2], Oksana Voloshanenko[2], Christian Hundsrucker[1], Mohammad J. Sadeh[1], Michael Boutros[2], Rainer Spang[1]

1 Department of Statistical Bioinformatics, Institute of Functional Genomics,
University of Regensburg, Germany
2 Division of Signaling and Functional Genomics, German Cancer Research Center (DKFZ) and Department of Cell and Molecular Biology, Faculty of Medicine Mannheim,
Heidelberg University, Germany

*** giusi.moffa@gmail.com**

S1 Protocols

Cell culture

Human colon cancer cells HCT116 were cultured upon standard conditioning using McCoy's medium (GIBCO) with 10% of fetal bovine serum. HCT116 TCF4/Wnt cells were cultured in the presence of 50 μg/ml hygromycin B.

Quantitative PCR (qPCR)

qPCR was performed as described in [1]. Briefly, cDNA was made with the help of the RevertAid H Minus First Strand cDNA Synthesis Kit (Thermo Fischer Scientific). Then cDNA was diluted and qPCR using the universal probe library system mix was made. UBC or GAPDH were used as calibrators.

Immunofluorescence

Immunofluorescence was performed according to the standard procedure. Pictures were taken with the confocal Leica SP5 LSM Microscope. The intensity of nuclear β-catenin staining was analyzed using the `R` package `EBImage` (`http://www.bioconductor.org/packages/2.2/bioc/html/EBImage.html`). The nuclear intensity of each cell was normalized to the median of the control cell population within one experiment and cells from three independent experiments were used to calculate p-values using the Mann-Whitney-Wilcoxon test.

RNAi Experiments

siRNAs against CTNNB1/β-catenin, Evi, APC, BCL9, TCF7L2, control were described in [1] and obtained from Ambion (Applied Biosystems, Foster City, CA). For RNAseq experiments cells were reverse transfected 6-well plate with $10nM$ siRNA using a total of 0.1%. DharmaFECT 1 (Thermo Fischer Scientific, in Waltham, MA). 72 hrs later cell were lysed and isolation of RNA was performed. For luciferase Wnt-reporter experiment cells were seeded into 384-well plate and 72 hrs after reverse transfection cells were lysed and D-lucifirin substrate was added. Subsequently, CTG (CellTiter-Glo® Luminescent Cell Viability Assay, Promega) was performed and luciferase signal was normalized to cell viability.

Expression profiling by RNA sequencing

Total RNA was extracted as described below and RNA integrity was verified on an Agilent 2100 Bioanalyzer. mRNA was isolated with the Dynabeads mRNA Purification Kit (Invitrogen). For each sample, a library for SOLiD sequencing was prepared using the 'Whole transcriptome analysis kit' (4409491 Rev. D, Applied Biosystems). In brief, polyA RNA was fragmented using RNAseIII, purified, hybridized and ligated to RNA adapters. Ligation products were reverse transcribed, purified and approx. 200 nt cDNA fragments were isolated after electrophoresis on a 6% TBE-Urea gel. cDNA was PCR amplified with SOLiD sequencing primers and purified to produce a library suitable as a template for emulsion PCR. DNA concentrations were determined using qPCR. A barcoded pooled library was generated by combining equimolar amounts from all samples. Emulsion PCR and sequencing with the SOLiD system was performed according to the manufacturers' protocol (Applied Biosystems).

Reads mapping

Mapping of SOLiD reads was performed using the mapReads algorithm, supplied by Applied Biosystems as part of the whole transcriptome analysis pipeline (v1.2). Two mismatches were allowed in both seed regions (bases 1-25, and bases 20-45) of the aligned read. Reads that mapped uniquely to the Human Genome (Hg18) were retained for further analysis. Reads were also mapped to a filter reference (Applied Biosystems). The filter library removed from further analysis reads that aligned to sequences from ribosomal RNA, tRNAs, single base repeats, adapter sequences. The python package HTSeq (v0.4.7), was used to count the number of reads mapping to each gene (`http://www-huber.embl.de/users/anders/HTSeq/doc/overview.html`). Expression values (read counts) were obtained by summing the number of reads that mapped uniquely to annotated regions of the Human Genome (Hg18). Reads that mapped to a locus that had more than one annotated gene were considered ambiguous and not used. The Bioconductor package `limma` was used to identify differentially expressed genes between siRNA knockdown samples and control siRNA. Differential expression was determined after preprocessing the read counts with the function `voom` to obtain weighted log count per million [2].

References

1. Voloshanenko O, Erdmann G, Dubash TD, Augustin I, Metzig M, Moffa G, et al. Wnt secretion is required to maintain high levels of Wnt activity in colon cancer cells. Nature communications. 2013;4.

2. Law CW, Chen Y, Shi W, Smyth GK. voom: precision weights unlock linear model analysis tools for RNA-seq read counts. Genome Biology. 2014;15:R29.
