# Supplementary material for: Refining Pathways: A Model Comparison Approach
Source: PLoS One. 2016 Jun 1;11(6):e0155999. doi: 10.1371/journal.pone.0155999 (PMC4889067; doi:10.1371/journal.pone.0155999)
Supplement: S1 Fig — Examples about the distribution of the sequence reads, with a quantification of the expression of both β-catenin alleles (mutated and wild-type) in HCT116 cells are reported in the supplementary material. (PDF) [file pone.0155999.s003.pdf]

Supplement- Predicting signalling pathway features by nested effect modelling, demonstrated on Wnt signalling in HCT116 cells

Giusi Moffa[1, *], Gerrit Erdmann[2], Oksana Voloshanenko[2], Christian Hundsrucker[1], Mohammad J. Sadeh[1], Michael Boutros[2], Rainer Spang[1]

1 Department of Statistical Bioinformatics, Institute of Functional Genomics,
University of Regensburg, Germany
2 Division of Signaling and Functional Genomics, German Cancer Research Center (DKFZ) and Department of Cell and Molecular Biology, Faculty of Medicine Mannheim,
Heidelberg University, Germany

*** giusi.moffa@gmail.com**

S1 Fig.

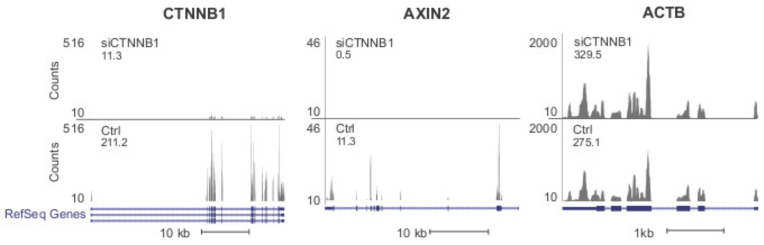

Sequence reads. Example plots of the distribution of reads for selected genes based on RNAseq experiments using *CTNNB1* (β-catenin) in the upper panels and control siRNAs in the lower panels. *AXIN2* is a target gene of canonical Wnt signalling, ACTB is an unrelated control. Sequence reads were mapped to the genome and the frequentcy at each base is plotted as height of the bars.
