# Supplementary material for: Refining Pathways: A Model Comparison Approach
Source: PLoS One. 2016 Jun 1;11(6):e0155999. doi: 10.1371/journal.pone.0155999 (PMC4889067; doi:10.1371/journal.pone.0155999)
Supplement: S2 Fig — Results from Western blot analysis are reported in the supplementary material. (PDF) [file pone.0155999.s004.pdf]

Supplement- Predicting signalling pathway features by nested effect modelling, demonstrated on Wnt signalling in HCT116 cells

Giusi Moffa[1, *], Gerrit Erdmann[2], Oksana Voloshanenko[2], Christian Hundsrucker[1], Mohammad J. Sadeh[1], Michael Boutros[2], Rainer Spang[1]

1 Department of Statistical Bioinformatics, Institute of Functional Genomics,
University of Regensburg, Germany
2 Division of Signaling and Functional Genomics, German Cancer Research Center (DKFZ) and Department of Cell and Molecular Biology, Faculty of Medicine Mannheim,
Heidelberg University, Germany

*** giusi.moffa@gmail.com**

S2 Fig.

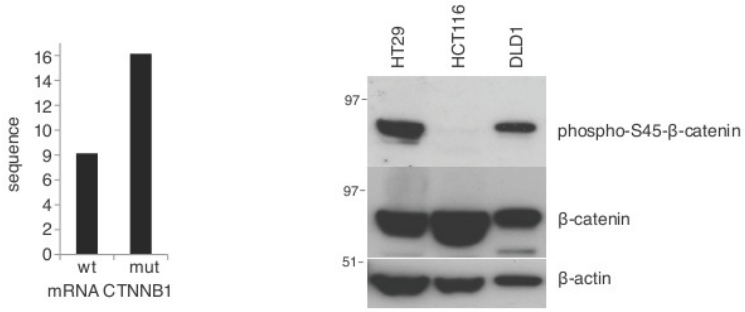

Phosphorylation of S45 of β-catenin is not detected in HCT116 cells. The left panel shows a quantification of the expression of both β-catenin alleles (mutated and wild-type) in HCT116 cells based on mRNA sequencing experiments. The figure reports on the cumulated counts from 4 independent sequencing runs. The right panel reports on the Western blot analysis. Cell lysates from the indicated cell lines were used for Western blot analysis with the indicated antibodies. One of 3 independent experiments is shown.
