# Supplementary material for: Refining Pathways: A Model Comparison Approach
Source: PLoS One. 2016 Jun 1;11(6):e0155999. doi: 10.1371/journal.pone.0155999 (PMC4889067; doi:10.1371/journal.pone.0155999)
Supplement: S1 Alternative Analysis — Details about the No-CONAN approach. (PDF) [file pone.0155999.s005.pdf]

Supplement - Refining pathways: a model comparison approach

Giusi Moffa[1, *], Gerrit Erdmann[2], Oksana Voloshanenko[2], Christian Hundsrucker[1], Mohammad J. Sadeh[1], Michael Boutros[2], Rainer Spang[1]

1 Department of Statistical Bioinformatics, Institute of Functional Genomics,
University of Regensburg, Germany
2 Division of Signaling and Functional Genomics, German Cancer Research Center (DKFZ) and Department of Cell and Molecular Biology, Faculty of Medicine Mannheim,
Heidelberg University, Germany

*** giusi.moffa@gmail.com**

S1 Alternative analysis

No-Conan on RNAseq expression data from the Wnt pathway in HCT116 cells

Hypothesis tests on the nested structure of the observed effects yield a partial learning of a signalling network

Recently, [1] developed an algorithm called No-CONAN for non confoundable network analysis, based on the idea of using a statistical test to decide whether pairs of variables should be connected or not. This type of approach is well established in the context of Gaussian graphical models and Bayesian networks for the reconstruction of a graph skeleton, where conditional independence tests are routinely used in constraint based learning methods. Connections which do not explain the observed data sufficiently well are rejected.

In order to gather insights about the directional flow and possibly the causal relations of the components of a pathway however it is useful to think of the gene signalling networks in terms of nested effects. No-CONAN builds on the idea of applying statistical tests to learn the structure of networks encoding nesting relationships between the effects E of the signalling nodes S. Assuming a deterministic nature of the signalling pathways and a binary measure of the effects (1 for present and 0 for absent), each topology defines a set of possible patterns which can be observed for the E-genes, under each intervention on the signal components. In the No-CONAN terminology configurations of the observed E-genes which are not compatible with the hypothesised underlying network are called alien patterns and can only be explained by noise.

No-CONAN focuses on pairs of pathway genes S_i and S_j and tests every possible up-/downstream relation between them, e.g. S_i is upstream of S_j, S_j is upstream of S_i, they are in a feedback loop, or they are unconnected. The tests are based on the nesting of downstream events in perturbation assays of S_i and S_j. Depending on its attachment position, each E-gene can show four different patterns, responding to silencing of either S-gene, to only one (one or the other) or to both at the same time. For example if we assume that S_i is upstream of S_j, observing that the targets of S_j do not show an effect when intervening on S_i would constitute an alien pattern. Similarly observing an effect on targets of S_i when intervening on S_j, and so on. If a test is significant it means that under the hypothesis that the tested connection is true, the observed pattern is unlikely

to be explained by noise alone, therefore the relation is discarded. The tests are applied to all possible pairs of perturbed S-genes.

Our RNAseq data covers only knockdown assays from 5 genes in the Wnt pathway. One might argue that this gives an incomplete account of this complex pathway and all its cross talk with other pathways. Although we do not make global claims about how the pathway works, one might still argue that the pathway needs to be modelled in its entirety, even if one only wants to decide on a single detail. Mechanisms beyond the modelled genes affect the data and might confound our interpretation of the data. Moreover, we only assess changes in the coding transcriptome of cells thus missing out on changes on other levels of cellular regulation like the non-coding transcriptome, chromatin structure, the proteome, or the metabolome. However, even in a huge project that collects all this information there could still be concerns that there are cellular mechanisms affecting our data and hence its interpretation that to date we do not even know of: the unknown unknowns of the pathway.

[1] have systematically addressed the problem of deciding which properties of an estimated network topology can be affected by unobserved or even unknown mechanisms and which cannot. Key to the algorithm is that hidden confounders do not influence the type of E-gene patterns which can be observed in response to each silencing experiment, leaving only the observational noise to explain the observation of alien patterns.

Every test has then the unique property that it cannot be confounded by hidden variables. The relation between a pair of S-genes would be fully resolved, if all but one relation were rejected. However, in many cases this is not possible due to the possible effects of hidden confounders that we need to account for. As explained in detail in [1], that rejection of the disconnected relationship between S_i and S_j is assumed in a strict sense, where no interaction between them are allowed even further downstream. In other words when the possibility of no connection is rejected for a pair of nodes, not only it is intended that there is no direct link between them, but also that they cannot have a common child. When considering two known signalling nodes S_i and S_j the relation $S_i \to H \leftarrow S_j$ of two nodes with a common child can explain every possible configuration of E-genes, therefore it is assumed a priori possible in the No-CONAN approach and can never be excluded. Likewise the only relation that can be fully resolved between two nodes is the topology with two parents and a common hidden child, in the case where the data reject every other possible relation. Interestingly the possibility to fully resolve networks of two parents with a common child means that the No-CONAN approach has the potential to identify the position of hidden nodes in a network. On the other hand it is clear that feedback loops of the type $S_1 \leftrightarrow S_2$ cannot be clearly identified since they include both relations $S_1 \to S_2$ and $S_2 \leftarrow S_1$, neither of which should be rejected if the feedback loop is the true underlying network. Nevertheless by excluding certain of the possible up/down-stream relations the No-CONAN approach provides us with a partially identified network topology a pNEM and important insights into which relations may be worth investigating further.

In general it is true for pNEM that the higher the number of relations which cannot be rejected the higher is the degree of uncertainty in the partially learned network. The limitation in learning is accepted in order to gain confidence in certain features which can be identified in a non confoundable manner. The edges in a pNEM describe relations which cannot be rejected from the observed data [1].

Technically No-CONAN performs a statistical test in order to detect unusually high numbers of observed alien patterns, which for a given connection are unlikely to be only due to noise. Let γ_R be a bound on the probability that alien patterns for relationship R are observed, it has been shown [1] that for each possible relation R between S_i and

S_j the probability of observing at least a alien patterns out of L can be bound as

$$P(A \geq a|R) = \sum_{l=a}^{L} \binom{L}{l} \gamma_R^l (1-\gamma_R)^{L-l}$$

and used to conduct a test, where a relationship R is rejected if

$$P(A \geq a|R) < \kappa$$

where κ is a calibration parameter typically set to .05.

Non confoundable network analysis rejects the dominant activation model using a statistical test that cannot be confounded by missing data or unknown unknowns of Wnt signalling

The difference between the DAM and the ASM is mainly captured by the type of edge between *EVI/WLS* and *CTNNB1/β-catenin*. Dominant activation assumes that this edge does not exist. It postulates that in CRC no information is flowing through the upstream parts of the Wnt signalling pathway and hence *EVI/WLS* is not on the path from the mutated proteins to the Wnt target genes. In contrast, activation by sensitisation postulates the autocrine stimulation of the intact Wnt pathway, and here *EVI/WLS* mediated secretion of Wnt ligands is an event that occurs upstream of the *CTNNB1/β-catenin* activity in the nucleus. Hence, in this model there must be an edge from *EVI/WLS* to *CTNNB1/β-catenin*. Our strategy is to use No-CONAN to test whether this edge exists or not. Since the No-CONAN tests are not confoundable by hidden data (no matter whether it was unobserved or observed but excluded from the analysis), we could confine the analysis to the RNAseq profiles from the *EVI/WLS* and *CTNNB1/β-catenin* knockdowns only. Since we are interested to learn whether in CRC *EVI/WLS* affects targets downstream of *CTNNB1/β-catenin* in the signalling we only included in the analysis E-genes that responded to the *CTNNB1/β-catenin* knockdowns.

Parameter calibration is critical to a No-CONAN analysis. The algorithm requires the input data to be in the form of a binary matrix $\boldsymbol{D} = (d_{lk})$ with rows D_l corresponding to E-genes and columns D_k corresponding to silenced S-genes. An element $d_{lk} = 1$ indicates that the expression of E-gene l was affected by the knockdown of S-gene k in the RNAi experiment. When testing the DAM model, it is assumed that there is no connection between *EVI/WLS* and *CTNNB1/β-catenin*. An unexpected or alien pattern is observed for E-genes which shows an effect both when perturbing *EVI/WLS* and β-catenin. No-CONAN tests whether the number of targets responding to both interventions is significantly higher than it would be expected by noise alone. No-CONAN requires an estimate of the size of the binary noise α to be expected in the data. This parameter is the probability of a false call and needs to be set by the user. The calibration of α can greatly affect the results and is therefore critical. For each knockdown the data were binarised on the basis of the posterior probability of differential expression. Values of the posterior probabilities above a certain threshold λ were set to 1 and values below λ were set to 0. Again the choice of an appropriate cutoff λ is critical to the analysis. Like in the previous analysis our strategy was to use multiple settings of the parameters to assess the robustness of the analysis. The discretising threshold was varied between .5 and .99 in steps of .01. Noise levels ranging from .02 and .32 were considered in the simulations. The p-value for rejecting the DAM, or more formally for rejecting the relation "unconnected" for the

pair of genes *EVI/WLS* and β-catenin, was found to be virtually zero for all noise levels, and no matter how many genes were included in the analysis.

The inclusion of all β-catenin responsive genes as E-genes might lead to the inclusion of genes that are not Wnt targets but are affected by β-catenin functions outside the pathway, e.g. in cell adherence mediated signalling. To filter those out, we rerun the analysis including only genes whose posterior probability of differential expression was above the cutoff λ for both the β-catenin and the *TCF7L2* knockdowns. Although the number of E-genes included in the analysis was greatly reduced, the No-CONAN again rejected the DAM over the full range of cutoff values and even for very high noise levels.

References

1. Sadeh MJ, Moffa G, Spang R. Considering Unknown Unknowns - Reconstruction of Non-confoundable Causal Relations in Biological Networks. Journal of Computational Biology. 2013;20:920–932.
